# Letter to the Editor

**Published:** 1986-08

**Authors:** D. J. Bracey

**Affiliations:** Consultant Orthopaedic Surgeon


### Letter to the Editor

Sir,

I was very interested to read Huw Griffith's contribution to the April BMCJ regarding the telephone transmission of X-rays and scans, as we used this equipment in Cornwall at an early stage in its development.

We were involved in a pilot study with the transmissions of X-rays from Casualty Departments in the Isles of Scilly and Penzance to the City Hospital in Truro in 1981. This was the first time the equipment had been used in clinical practice in this country. I presented the results of our first 18 months' experience to the South West Radiologists' Association on the 9th October 1982. From July 1981 to September 1982 a total of 73 transmissions was made. Fifteen transmissions failed for a number of reasons including failure to remove the lens cover from the video camera at the transmitting end (!).

The equipment has since been considerably improved and the Imtran system which is now permanently set up between Penzance and Truro has proved to be much more reliable.

D. J. Bracey
Consultant Orthopaedic Surgeon

